# Extracellular vesicles: A potential future strategy for dental and maxillofacial tissue repair and regeneration

**DOI:** 10.3389/fphys.2022.1012241

**Published:** 2022-11-21

**Authors:** Ziwei Liu, Situo Wang, Na Huo, Shuo Yang, Quan Shi, Juan Xu

**Affiliations:** ^1^ Medical School of Chinese PLA, Beijing, China; ^2^ Department of Stomatology, The First Medical Center, Chinese PLA General Hospital, Beijing, China; ^3^ Orthopedic Laboratory of PLA General Hospital, Beijing, China

**Keywords:** extracellular vesicles, regenerative medicine, dental and maxillofacial tissue repair, therapeutic effects, clinical application

## Abstract

Extracellular vesicles (EVs), nano-sized bilayer membrane structures containing lipids, proteins and nucleic acids, play key roles in intercellular communication. Compared to stem cells, EVs have lower tumorigenicity and immunogenicity, are easier to manage and cause fewer ethic problems. In recent years, EVs have emerged as a potential solution for tissue regeneration in stomatology through cell-free therapies. The present review focuses on the role of EVs in dental and maxillofacial tissue repair and regeneration, including in dental and periodontal tissue, maxilla and mandible bone, temporomandibular joint cartilage, peripheral nerve and soft tissue. We also make a brief overview on the mechanism of EVs performing functions. However, limitations and challenges in clinical application of EVs still exist and should be addressed in future researches.

## 1 Introduction

Damage or defects in dental and maxillofacial tissue are usually caused by various factors, such as trauma, infection, inflammation, and tumors. Severe tissue defects will bring substantial pain for patients and seriously affect their quality of life. At present, oral and maxillofacial tissue defects are often directly filled with exogenous materials or replaced with maxillofacial prostheses. However, neither structure nor function can be attained in this way. Achieving physiological repair and the true regeneration of dental and maxillofacial tissues has become a research focus.

Over the past two decades, numerous preclinical and clinical studies have demonstrated the critical role of mesenchymal stem cells (MSCs) in tissue engineering and regenerative medicine, due to their self-renewal, multidirectional differentiation and immune regulation capabilities. These characteristics are shared by different types of oral MSCs, such as dental pulp stem cells (DPSCs) (Zhao et al., 2012; Di Tinco et al., 2021), gingival mesenchymal stem cells (GMSCs) (Nakao et al., 2021) and stem cells from exfoliated deciduous teeth (SHEDs) (Miura et al., 2003). Although the therapeutic effect of MSCs for the treatment of oral diseases has been affirmed, there are certain problems caused by cell therapy. Therefore, as the main component of cell paracrine signaling, extracellular vesicles (EVs) have attracted more attention from scholars.

EVs are membrane vesicles secreted by different types of cells, including immunocytes, blood cells, and MSCs, and have been extracted from a wide range of body fluids including urine, breast milk, saliva, synovial fluid, and plasma (Yáñez-Mó et al., 2015). EVs contain proteins, lipids, nucleic acids (miRNA, mRNA, and DNA), or other bioactivators (Colombo et al., 2014), depending on the type and differentiation status of the parent cells and the stimulating factors in the microenvironment. After interacting with the target cell under certain conditions, the contents of the EVs are released into the target cell, thereby mediating intercellular communication and exerting biological functions (Maia et al., 2018).

EVs are roughly divided into three subtypes according to their size and biogenesis: exosomes, microvesicles, and apoptotic bodies (Théry et al., 2018). Exosomes originate from the endosomal system by inward budding of late endocytic compartments, and have a double-layer lipid structure with a diameter ranging from 30 to 150 nm. Microvesicles (MVs) are regenerated from direct budding of the plasma membrane, with a diameter of approximately 100–1,000 nm. Apoptotic bodies are generally produced during the process of apoptosis, with a diameter of approximately 1–5 μm (van Niel et al., 2018). The formation of apoptotic bodies results from cell decomposition, which is a complex process that varies among types of cells; thus, the cellular components of apoptotic bodies are also different (Díaz-Flores et al., 2018). Different types of EVs have different characteristics based on their distinct contents. The sorting and packaging of EVs cargo are a complex and multi-staged process rather than a random grab (Elsharkasy et al., 2020). Several mechanisms may play a role: 1) Autologous protein composition of EVs affects its biogenesis and release, such as components of the endosomal sorting complex for transport (ESCRT), Rab GTPases, annexin and flotillin (Gutiérrez-Vázquez et al., 2013; Villarroya-Beltri et al., 2014). 2) Some RNA binding proteins are involved in the sorting of miRNA in EVs, which is regulated by specific sequence motifs (Villarroya-Beltri et al., 2013; Santangelo et al., 2016). 3) EVs may contain proteins capable of binding and sorting its own mRNA, such as Arc (Pastuzyn et al., 2018). Researchers have demonstrated that EVs participate in most physiological or pathological processes in cells (You et al., 2020), including signal transduction (Codagnone et al., 2017), cell growth and development (Zheng et al., 2020), metabolism (Akbar et al., 2019), and immune responses (Szekeres-Bartho et al., 2018). The advantages of EVs also include effectively reducing the possibility of tumorigenesis, immunological rejection and ethical issues caused by cell therapy. Moreover, their effectiveness lasted for approximately 6 months under appropriate storage conditions (Yu et al., 2014). Therefore, the application of EVs is expected to prevent the adverse consequences of cell therapy in oral and maxillofacial tissue repair and promote the development of regenerative medicine.

**FIGURE 1:**
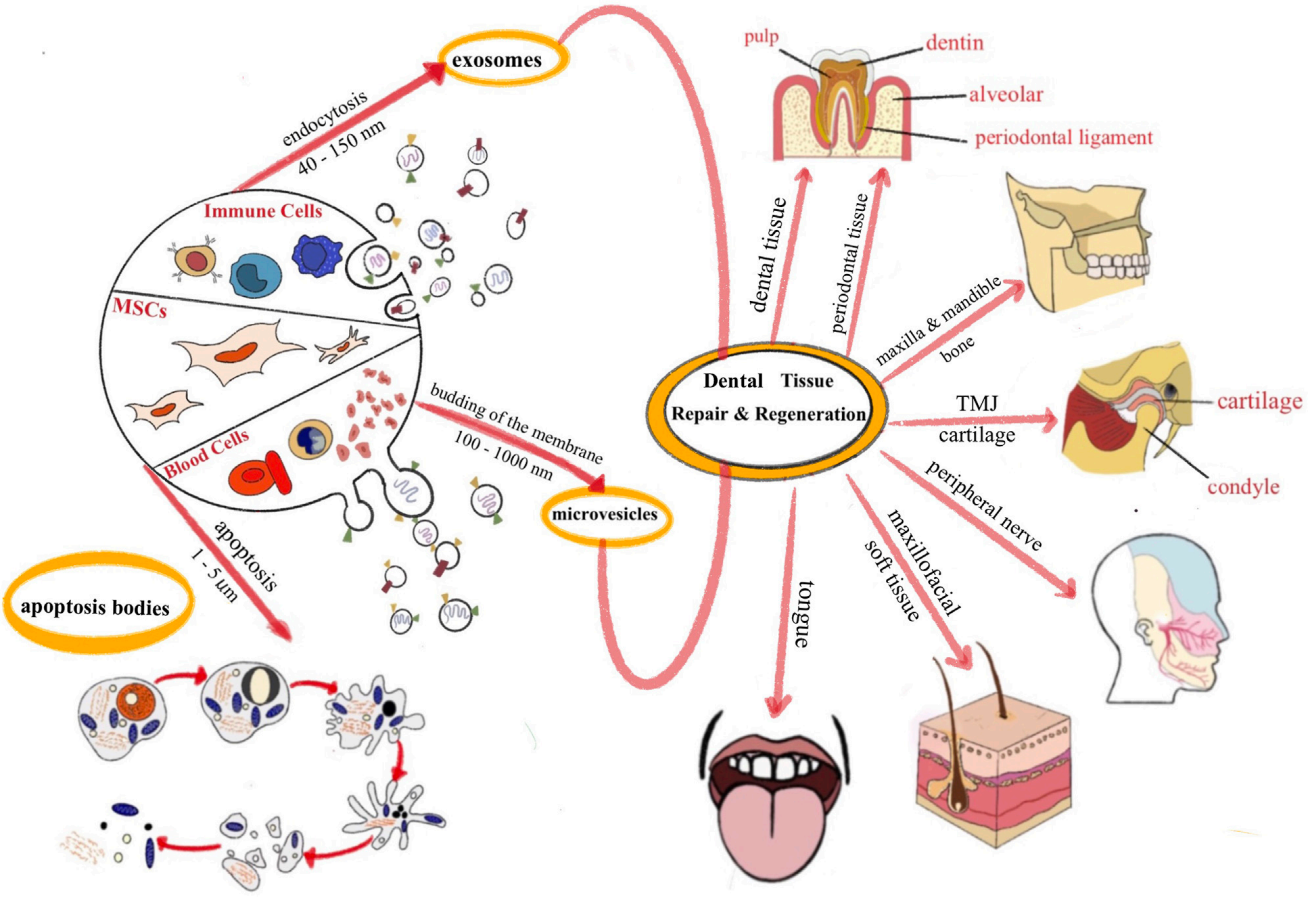
The application of EVs for dental and maxillofacial tissue repair and regeneration.

In summary, due to the limitations of stem cell therapy, cell-free therapy has become a research focus for the physiological repair and regeneration of defective tissue. Using different EV sources to repair different tissue types is a sensible strategy to achieve better effects. The use of EVs to repair oral and maxillofacial tissue defects is reviewed in more detail below (Figure 1).

## 2 The use of EVs in oral and maxillofacial tissue repair and regeneration

### 2.1 Dental tissue

Dental tissue includes enamel, dentin, pulp and cementum. Pulp, a critical structure rich in cells, blood vessels and nerves, is protected by mineralized enamel and dentin. If caries is not treated properly and timely, bacteria will easily enter the pulp system and affect pulp tissue, leading to irreversible pulpitis or pulp necrosis that requires root canal treatment. However, after the treatment, teeth lose their nutrient supply and are prone to discoloration or even fracture, which is not conducive to maintaining oral cavity health. To achieve pulp tissue regeneration, regenerative endodontic procedures have been proposed as an alternative to conventional pulp treatment.

#### 2.1.1 Pulp

According to some researchers, the core problem of pulp regeneration is whether there is a healthy pulp stump or residual pulp stem cells. Otherwise, the new growth of tissue is not true pulp regeneration but the replacement of pulp with new periodontal tissue (Lui et al., 2020). EVs directly promote the proliferation, migration, odontogenic differentiation and revascularization of pulp stem cells or precursor cells, either of which can speed up pulp regeneration. A study showed that EVs derived from stem cells from the apical papilla (SCAPs) promoted the migration, proliferation and differentiation of endogenous stem cells around the root apex, thereby promoting pulpo-dentinal complex regeneration (Zhuang et al., 2020). Endogenous stem cells refer to mesenchymal stem cells from other body locations; for example, human bone marrow mesenchymal stem cells (BMMSCs) were recruited to the root apex and became an important source of stem cells promoting pulp regeneration (Ivica et al., 2020). Huang et al. demonstrated that exosomes isolated from dental pulp stem cells (DPSC-Exos) increased the secretion of pulp-like tissue by naïve DPSCs and suggested that odontogenic differentiation was promoted by the transfer of microRNAs (miRNAs) in exosomes (Huang et al., 2016). In addition, DPSC-Exos also induced blood vessel regeneration *in vitro* by promoting the proliferation of endothelial cells and increasing the expression of angiogenic factors (Xian et al., 2018).

#### 2.1.2 Dentin

Dentin is formed by the mineralization of the matrix secreted by odontoblasts. During caries development, a series of autocrine and paracrine signals induce teeth to produce reparative dentin, prevent caries development, and resist pulpo-dentinal complex infection (Couve et al., 2018).

Pulp stem cells reside in the pulp cavity. These cells grow rapidly and can be induced by multiple factors to form odontoblasts and secrete dentin matrix. Swanson et al. utilized the intercellular communication properties of EVs derived from DPSCs to catalyze the formation of a reactionary dentin bridge by recruiting endogenous dental stem cells and upregulating odontogenic gene expression, thereby increasing mineralization *in vitro* (Swanson et al., 2020). Neural crest-related Schwann cell precursors (SCPs) are an important cellular source of dental MSCs, and able to migrate to the injured site, differentiate into odontoblasts and dental pulp cells (Kaukua et al., 2014). It is found that DPSCs share a common lineage with neural crest-derived SCPs and have neural crest stem features, so they can differentiate to both Schwann cell (SCs) and odontoblast lineages (Uribe-Etxebarria et al., 2017; Luzuriaga et al., 2019). Moreover, DPSC-Exos, especially exosomes derived from lipopolysaccharide (LPS)-pretreated DPSCs, play a critical role in promoting the proliferation, migration and odontogenic differentiation of SCs (Li et al., 2021).

#### 2.1.3 Cementum

Cementum is a thin layer on the root surface that serves as an antiresorptive barrier and anchorage for a functional periodontal ligament (Feller et al., 2016), which is paramount for periodontal regeneration. Extensive literature about stem cells promoting cementum repair has been published. For example, periodontal ligaments mesenchymal stem cells (PDLSCs) are the most favorable stem cell population utilized in periodontal regeneration, due to their high expression of cementoblastic/osteoblastic markers, including alkaline phosphatase, MEPE, bone sialoprotein, osteocalcin, and TGFβ receptor type I, which are responsible for the formation of the cementum-periodontal ligament complex (Seo et al., 2004). Dental Follicle Stem Cells (DFSCs) were also demonstrated to form PDL-like structures or calcified nodules with cementum-like structures, which suggested their potential for periodontal differentiation (Han et al., 2010). A recently published paper showed that the effects of macrophages on cementoblast mineralization were mediated by exosomes. Compared with M0 (unpolarized macrophages), M1-polarized macrophages attenuated cementoblast mineralization, while M2-polarized macrophages enhanced cementoblast mineralization (Zhao et al., 2022). However, there are few articles about EVs promoting cementum regeneration.

### 2.2 Periodontal tissue

Periodontitis is considered as a global epidemic. Long-standing pathological factors may destroy the supporting periodontal tissue and result in tooth loss. Conventional periodontal treatment can successfully reduce the number of pathogens in periodontal defects. However, no effective methods for regenerating and reconstructing lost tissues have been developed. Since the inflammatory microenvironment caused by periodontitis is the main cause for the abnormal function and regenerative defects of local stem cells, the progression of periodontitis and disease severity is closely related to the host’s immune response. Therefore, many researchers have begun to promote periodontal tissue regeneration by reducing inflammation and mediating immunoregulation.

#### 2.2.1 Alveolar bone

Many studies have shown that EVs increase the bone regenerative capacity of stem cells in periodontitis tissues and alleviate the inhibitory effects of local stem cells caused by inflammation. Research has shown that human periodontal ligament cells (hPDLCs) cultured in conditioned medium of BMMSCs increased expression of bone formation-related genes and proteins (Shyh-Chang and Ng, 2017), which indicates that BMMSC-EVs promoted odontogenic differentiation in hPDLCs. BMMSC-EVs have therapeutic effects on rats with periodontitis. This effect may partly be mediated by BMMSC-EVs-mediated activation of the OPG-RANKL-RANK signaling pathway that regulates the function of osteoclasts and promotes TGF-β1 expression and macrophage M2 polarization, thus inhibiting periodontitis and alveolar bone absorption (Liu et al., 2021). In addition, with powerful anti-inflammatory and immunomodulatory effects, DPSC-Exos effectively promoted alveolar bone reconstruction and periodontal epithelial healing in a mouse model through the exosome-mediated transmission of miR-1246 (Shen et al., 2020). It is also known that the accumulation of intracellular ROS, a mediator of mitogen-activated protein kinase (MAPK) family members, affects MAPK/JNK signaling pathways, which can be inhibited by antioxidants to reduce ROS accumulation (Yang et al., 2018). A recent study showed that in an inflammatory microenvironment, ROS levels were significantly reduced in PDLSCs after being treated with LPS-preconditioned DFSC-EVs, which possibly exhibited their antioxidant effects through the ROS/MAPK signaling pathway, thereby treating periodontitis (Huang et al., 2022).

#### 2.2.2 Periodontal ligament

The density and directional arrangement of fibers in the periodontal ligament (PDL) are closely related to the growth of the cementum, the distribution of masticatory force and the remodeling of alveolar bone. Adipose stem cells (ADSCs) were demonstrated to promote the formation of a new functional PDL by producing more oblique and horizontal fibers with an ideal arrangement and direction (Monsarrat et al., 2014). EVs from adipose stem cells (ADSC-EVs) were injected into the ligature-induced rat periodontal pocket, and then well-organized periodontal tissue was observed perpendicular to the cementum and alveolar bone. Thus, ADSC-EVs enhanced the reconstruction of collagen fibers during PDL wound healing. Moreover, the therapeutic effect of ADSC-EVs was superior to that of ADSCs alone; the former induced a significantly larger newly formed periodontal tissue area (Mohammed et al., 2018), which may be relevant to the different metabolic processes associated with EVs and stem cells in the body. Another study demonstrated that MSC-EVs activated CD73-mediated adenosine receptor activation of the prosurvival AKT and ERK signaling pathways to promote the proliferation and migration of PDLCs, enhance cell viability, improve periodontal ligament function and promote tissue regeneration in a rat periodontal ligament defect model (Chew et al., 2019).

### 2.3 Temporomandibular joint cartilage

The temporomandibular joint comprises the mandibular condyle, articular facet, articular disc, articular capsule and articular ligaments. The cartilage on the joint surface has a strong protective effect on the bone. Due to the limited self-healing capacity of joint cartilage, temporomandibular joint osteoarthritis (TMJOA) often occurs after excessive wear (Zhao and Xie, 2021). The main treatment strategy is to inhibit the inflammatory response, prevent the gradual destruction of cartilage and subchondral bone, induce cartilage regeneration, and restore function (Wang et al., 2015).

The key to repairing cartilage defects is regulating the inflammatory response to prevent cartilage damage, promote the proliferation and migration of cartilage cells, and increase cartilage matrix synthesis. MiR-100, the product of EVs derived from SHEDs, inhibited the expression of proinflammatory factors (such as IL-6 and IL-8) and matrix metalloproteinases (such as MMP1 and MMP-9), thereby reducing inflammation in the temporomandibular joint and preventing further cartilage damage (Luo et al., 2019). Researchers established a rabbit TMJOA model and found that human umbilical cord blood mesenchymal stem cells (UCB-MSCs) demonstrated robust potential for cartilage protection and regeneration. MSC-EVs also promoted the recovery of TMJOA by regulating inflammation (Kim et al., 2019). In addition, MSC-EVs were proved to enhance the repair of critical-sized osteochondral defects in an adult immunocompetent rat model, and the new hyaline cartilage and underlying subchondral bone closely resembled normal tissue (Zhang et al., 2016). Researchers have suggested that this outcome could be attributed to exosomal CD73-mediated adenosine activation of AKT, ERK, and AMPK signaling, which increases chondrocyte activity and induces cell proliferation and tissue regeneration through kinase phosphorylation (Zhang et al., 2018).

### 2.4 Maxillofacial soft tissue

In recent years, soft tissue replacement has been increasingly demanded for treating congenital malformation, traumatic repair and postoperative rehabilitation of maxillofacial tumors. Researchers are seeking a stem cell-based therapy or cell-free approach to producing engineered adipose tissue to develop an ideal soft tissue substitute. Exosome-like vesicles derived from adipose tissue induced adipogenesis in ADSCs and promoted the proliferation, migration, and angiogenic potential of aortic endothelial cells (Dai et al., 2017). MiR-450a-5p carried by EVs derived from adipose tissue triggered adipogenic signals in ADSCs by inhibiting the expression of WISP2 and promoting the differentiation of adipose cells (Zhang et al., 2017). In recent years, EVs have also been widely studied in the context of skin damage repair, but there have been few reports on the repair of maxillofacial skin. The main mechanism by which skin defects are repaired has been verified in different phases. For example, ADSC-EVs exerted anti-inflammatory effects by eliciting macrophage switching from an M1 to an M2 phenotype during the inflammatory phase (Lo Sicco et al., 2017), while EVs derived from UCB-MSCs stimulated wound healing by reducing scar formation in the remodeling stage (Zhang et al., 2021). In the proliferative phase, critical steps involve promoting the proliferation and migration of human dermal fibroblasts and human keratinocytes, as well as inducing angiogenic activity in endothelial cells (Zeng and Liu, 2021). Exosomes from human endometrial MSCs promoted the expression of angiogenic markers and the proliferation and migration of human umbilical vein endothelial cells (Ha et al., 2020).

### 2.5 Peripheral nerves

Peripheral nerve injuries typically lead to motion and sensory dysfunction in different parts of the body. For example, the trigeminal and facial nerves are easily affected by maxillofacial tumors or severe trauma, causing damage to facial sensation and even leading to facial paralysis. Nerve regeneration after injury is a complex process involving the differentiation of SCs, the recruitment of macrophages, the growth of blood vessels and the regeneration of axons (Hercher et al., 2022). Interestingly, dental MSCs, which derived from the neural crest during embryonic development, show stronger nerve regenerative and neuroprotective effects than MSCs derived from other sources (Ibarretxe et al., 2012; Mayo et al., 2014). Furthermore, a study compared three types of dental MSCs (follicle, papilla, and pulp) from a single donor and found that MSCs derived from pulp showed higher neurogenic potential than those from the follicle and papilla (Ullah et al., 2016). Application of conditioned media (CM) or concentrated EVs secreted by dental stem cells have also been found to facilitate nerve repair and regeneration. With a model of polyneuropathy in diabetic rats, DPSC-conditioned media (DPSC-CM) were found to promote neurite outgrowth of dorsal root ganglion neurons and increase the viability and myelin-related protein expression of SCs (Omi et al., 2017). The gene expression of transient receptor potential vanilloid channel-1 (TRPV1), a pain receptor thought to be related to the regeneration of peripheral nerves, can be upregulated after nerve injury, and a TRPV1 antagonist can inhibit the injury process and promote axon regeneration (Ren et al., 2015). However, researchers found that DPSC-CM upregulated neuron-related markers and the gene expression of TRPV1, and promoted the survival and regeneration of isolated primary trigeminal ganglion neuronal cells (TGNCs), thereby repairing damaged trigeminal nerves (Sultan et al., 2020). GMSC-EVs effectively promoted the proliferation and migration of SCs, upregulated the expression of genes associated with the SC repair phenotype, and activated this phenotype to promote the regeneration of peripheral nerves (Mao et al., 2019). In addition, the nerve repairing effect of EVs from other source of cells has also been reported, such as SCs (Ching et al., 2018) or macrophages (Qing et al., 2018) derived EVs. Mechanical stimulation affected intercellular communication between neurons and SCs by changing the miRNA composition of SC-EVs. EVs produced by mechanical stimulation of SCs transferred miR-23b-3p from SCs to neurons, downregulated the expression of neuropilin 1, and promote the repair and regeneration of damaged peripheral nerves to some extent (Xia et al., 2020).

### 2.6 Tongue

Morbidity associated with oral cancers is high worldwide, and the tongue is the most vulnerable tissue. Currently, the standard method for treating oral cancer is surgical resection of the malignant tumor and involved tissue and reconstruction of the defective tissue with a free flap. Subsequently, postoperative chemotherapy is usually performed. However, using tissue flaps to rebuild the tongue has difficulty in functional restoration, especially taste bud regeneration in the reconstruction area. Thus, this treatment seriously affects patients’ prognosis and quality of life (Mannelli et al., 2018). Therefore, regenerating the lingual papilla and taste buds is the key to successfully rebuilding the tongue. By establishing a critical-sized tongue defect model in rats and combining the transplantation of small intestinal submucosa–extracellular matrix (SIS-ECM) with GMSC-EVs, researchers found that local implantation of exosome/SIS-ECM significantly increased the expression of CK14 and CK8 compared to the SIS-ECM control group, thus enhancing the regeneration of epithelial progenitor cells and facilitating taste bud and lingual papilla restoration (Zhang et al., 2019). Although studies of EV repair of the tongue are rare and have certain limitations, the results suggest that EVs are promising for clinical tongue reconstruction and taste bud regeneration after tongue carcinoma resection (The effects of EVs in dental and maxillofacial tissue repair and regeneration are listed in Table 1).

**TABLE 1:** Outlines of the effect of EVs in dental and maxillofacial tissue repair and regeneration approaches.

Target tissue	EV source	Cargo	Mechanism	Result	Ref.
Pulp	SCAPs		enhance DSPP expression	promoted dentine-pulp complex regeneration	Zhuang et al. (2020)
	DPSCs		trigger the P38 mitogen-activated protein kinase pathway	triggered regeneration of dental pulp-like tissue	Huang et al. (2016)
	DPSCs		promote the expression levels of MMP-9, VEGF-A, and KDR	induce blood vessel regeneration	Xian et al. (2018)
Dentin matrix	DPSCs		stimulate the migration of endogenous DPSCs and guide their differentiation toward secretory odontoblasts	induce tertiary dentin bridge formation	Swanson et al. (2020)
	LPS-pretreated DPSCs		facilitate SCs migration and odontogenic differentiation		Li et al. (2021)
	DPSCs		promote the odontogenic differentiation of DPSCs and improve hydroxyapatite nucleation	improve the formation and mineralization of dentin matrix	Chen et al. (2021)
Cementum	M0/M1/M2 macrophages		mediated by secreted substances from macrophages and was conducted through the culture fluid to the cementoblasts.	M1-polarized macrophages attenuated cementoblast mineralization, while M2-polarized macrophages enhanced cementoblast mineralization	Zhao et al. (2022)
Alveolar bone	BMMSCs		activate the OPG-RANKL-RANK signaling pathway	inhibit periodontitis and alveolar bone absorption	Liu et al. (2021)
	DPSCs	miR-1246	facilitate macrophages to convert from a pro-inflammatory phenotype to an anti-inflammatory phenotype	promote alveolar bone reconstruction and periodontal epithelial healing	Shen et al. (2020)
	LPS-preconditioned DFSCs		inhibit ROS/JNK signaling pathway under inflammatory conditions and promote macrophages to polarize toward the M2 phenotype *via* ROS/ERK signaling	enhance the therapeutic efficacy for periodontitis	Huang et al. (2022)
Periodontal ligament	ADSCs		enhance the proliferation of primitive periodontal fibroblast and osteoid tissues	enhance the outcome of the nonsurgical periodontal treatment	Mohammed et al. (2018)
	MSCs		through adenosine receptor activation of AKT and ERK signaling pathways	promote periodontal regeneration with enhanced bone growth and increased functional PDL length	Chew et al. (2019)
Temporomandibular joint cartilage	SHEDs	miR-100	inhibit the expression of proinflammatory factors and matrix metalloproteinases	reduce inflammation in the temporomandibular joint and prevent further cartilage damage	Luo et al. (2019)
	hUCM-MSCs		upregulate expression of growth factors, extracellular matrix markers, and anti-inflammatory cytokines, and reduce expression of pro-inflammatory cytokines	has potential for cartilage protection and cartilage regeneration	Kim et al. (2019)
	human embryonic MSCs			display complete restoration of cartilage and subchondral bone	Zhang et al. (2016)
	human embryonic MSCs	CD73	activate AKT and ERK phosphorylation and display a regenerative immune phenotype	mediate the repair of osteochondral defects	Zhang et al. (2018)
Maxillofacial soft tissue	adipose tissue		promote proliferation, migration, and angiogenesis of ECs and induce adipogenesis of ADSCs	induce adipose tissue regeneration	Dai et al. (2017)
	adipose tissue	miR-450a-5p	inhibit the expression of WISP2 and promote the differentiation of adipose cells	trigger fat-generating signals in adipose stem cells	Zhang et al. (2017)
	human endometrial MSCs		increase the expression of angiogenic markers and promote the proliferation and migration of HUVECs	induce angiogenic activity in endothelial cells	Ha et al. (2020)
Peripheral nerve	DPSCs		promote neurite outgrowth of DRG neurons and increase the viability and myelin-related protein expression of Schwann cells	contribute to the neurophysiological and neuropathological recovery	Omi et al. (2017)
	DPSCs		upregulate neuron-related markers and the gene expression of TRPV1 and promote the survival and regeneration of isolated primary TGNCs	repair damaged trigeminal nerves	Sultan et al. (2020)
	GMSCs		activate c-JUN-governed repair phenotype of SCs	promote peripheral nerve regeneration	Mao et al. (2019)
	Schwann cells	miR-23b-3p	downregulate the expression of Nrp1	promote the repair and regeneration of damaged peripheral nerves	Xia et al. (2020)
Tongue	GMSCs		increase the expression of CK14 and CK8, enhance the regeneration of epithelial progenitor cells	facilitate taste bud and lingual papilla restoration	Zhang et al. (2019)

## 3 Mechanism by which EVs promote dental and maxillofacial tissue repair and regeneration

Cell-to-cell communication involving exosome-borne cargo, such as miRNA, has emerged as a critical mechanism for tissue repair. Moreover, EVs can be taken up by various cells such as tissue cells, immune cells, and vascular endothelial cells, thus affecting the tissue repair process in multiple ways. This multifactor synergistic effect may be the key to the strong ability of MSC-EVs to promote tissue regeneration.

### 3.1 Enhancing proliferation, migration, and differentiation of tissue cells

MSCs regulate the biological properties of local tissue cell by releasing EVs, thereby maintaining tissue homeostasis and regeneration. Regarding wound healing, EVs enhanced the proliferation and migration of keratinocytes, fibroblasts, and endothelial cells (Tutuianu et al., 2021). This effect is often accompanied by upregulation of the PI3K-AKT-mTOR-HIF-1α signaling pathway and blocked by PI3K-AKT inhibitor both *in vitro* and *in vivo* (Liu et al., 2021). Another study showed that exosomes mediated the crosstalk between keratinocytes and macrophages in cutaneous wound healing as impairment in miRNA packaging of keratinocyte exosomes inhibited inflammation resolution and compromised functional wound closure (Zhou et al., 2020).

EV-mediated cellular communication between osteoblasts and osteoclasts is a newly discovered mechanism that regulates bone reconstruction. Osteoblasts release EVs containing RANKL and transfer them to the precursor cells of osteoclasts, which then differentiate into osteoclasts by stimulating the OPG/RANKL/RANK signaling pathway, which is critical in bone metabolism (Huynh et al., 2016). Exosomes secreted by SHEDs (SHED-Exos) effectively promoted human umbilical vein endothelial cells (HUVECs) blood vessel formation and osteogenic differentiation in BMMSCs. These effects can be suppressed by an AMPK inhibitor, suggesting that SHED-Exos contribute to bone regeneration by promoting neovascularization and new bone formation through the AMPK signaling pathway (Wu et al., 2019; Liu et al., 2020). In addition, increasing evidence suggests that apoptosis is a crucial link in tissue repair and regeneration, but the exact functions of apoptotic events remain largely unclear (Li et al., 2020). Apoptosis is closely related to cell proliferation (Pellettieri et al., 2010), differentiation (Hristov et al., 2004), and elimination of senescent cells (Jeon et al., 2017), and is essential for the maintenance of normal tissue homeostasis (Gaipl et al., 2005). During apoptosis, BMMSCs produce large amounts of apoptotic cell-derived extracellular vesicles. These EVs activate JNK signal transduction by increasing intracellular reactive oxygen species levels, effectively promoting the proliferation, migration, and osteogenic differentiation of BMMSCs and accelerating new bone formation (Li et al., 2022).

### 3.2 Preventing apoptosis of tissue cells

Despite promoting cell proliferation, migration and differentiation, EVs also facilitate the survival of recipient cells. Under pathological conditions such as hypoxia and inflammation, MSC-EVs promote tissue regeneration by maintaining tissue cell survival and preventing apoptosis. For example, EVs suppressed the inflammatory immune microenvironment, which is often enriched by proinflammatory immune cells and cytokines that reduce chondrocytes apoptosis (Yin et al., 2022). Another study revealed that hucMSC-EVs inhibited chondrocyte apoptosis by lowering the m6A level of NLRP3 mRNA with miR-1208 targeting combined with METTL3 (Zhou et al., 2022).

Neurons are a special kind of tissue cells and EVs can also promote nerve regeneration by inhibiting neuronal apoptosis. Researchers have found that BMMSC-EVs prevented hippocampal neuron apoptosis induced by oxygen and glucose deprivation by regulating the JMJD3/p53/KLF2 axis, which caused miR-93 to be transferred into hippocampal neurons (Luo et al., 2022). MSC-EVs reduced apoptosis and inflammation, and promoted angiogenesis after spinal cord injury, and miR-21-5p was one of the most highly expressed miRNAs in these EVs. Moreover, inhibition of miR-21-5p in MSC-EVs significantly reversed the beneficial effects of EVs on motor function and apoptosis, an effect that was associated with modulating FasL expression (Zhou et al., 2019).

### 3.3 Increasing mineralization

Mineralization is one of the most important steps in repairing and regenerating teeth, alveolar bone and the jawbone. Exogenous administration of osteogenic exosomes increased BMMSC mineralization in a dose-dependent manner, and the increase in mineralization was accompanied by upregulation of the expression of osteogenic markers including osteocalcin, bone sialoprotein and RUNX family transcription factor 2 (RUNX2), and increased protein secretion of the same gene products (Swanson et al., 2020). A study showed that mineralizing preosteoblast MC3T3-E1 exosomes had a significant promotion effect on bone marrow stromal cell osteogenic differentiation, as manifested by upregulated expression of the osteogenic marker genes RUNX2 and ALP, as well as enhanced matrix mineralization (Cui et al., 2016). In addition, during dentin formation, calcium mobilization and Ca^2+^ entry across the plasma membrane by odontoblasts are key physiological events. STIM1 is a Ca^2+^ concentration sensor located in the endoplasmic reticulum. When STIM1 expression is upregulated, Ca^2+^ is transferred to the extracellular matrix (ECM), and DPSCs release more EVs carrying minerals and organic matrix. These EVs are capable of promoting the odontogenic differentiation of DPSCs and improving hydroxyapatite nucleation, thus playing a key role in the differentiation of pulp stem cells and the formation and mineralization of dentin matrix (Chen et al., 2021).

### 3.4 Promoting angiogenesis

Blood vessels transmit mineral substances, growth factors and various progenitor cells to corresponding regions to participate in regenerative activity and help maintain homeostasis. Rapid vascular regeneration and reconstruction provide sufficient oxygen and nutrients for tissue and cells (Grosso et al., 2017). Angiogenesis is also a dynamic process based on vascular endothelial cells (ECs) and their surrounding environment. EC proliferation, migration, and tube formation are the foundations of vascularization (Lv et al., 2020). Liu et al. found that EVs derived from human induced pluripotent stem cells promoted the proliferation, migration and angiogenesis of vascular endothelial cells by activating the PI3K/Akt pathway (Liu et al., 2017). MiR-126, miR-130a, and miR-132 in EVs also play important roles in regulating these processes (Zhu et al., 2018). Huang et al. found that LPS-pretreated exosomes from DPSCs changed the expression profiles of miRNAs and activated MAPK signaling by increasing the expression of Kinase insertion domain receptor (KDR/VEGFR2), and also raised the levels of vascular endothelial growth factor (VEGF) expression, which eventually promoted the proliferation, migration and tube formation capacity of HUVECs *in vitro* (Huang et al., 2021). In addition, vascular damage through different mechanisms can also be inhibited or even reversed by MSC-derived exosomes. Exosomes play a critical role in repairing DNA double-strand breaks and alleviating oxidative damage. Exposure to MSC exosomes reduced apoptosis caused by radiation-induced DNA damage in vascular endothelial cells (Wen et al., 2016).

### 3.5 Anti-inflammatory effects and immunoregulation

EVs have strong anti-inflammatory and immunoregulatory effects. Researchers have shown that MSC-Exos combined with functionalized scaffolds induced innate and adaptive immunomodulatory responses to guide the biological communication network (Su et al., 2021). The number, size and biologically active material of EVs are altered in numerous inflammatory conditions. Moreover, EVs affected the cellular functions of neutrophils, monocytes, macrophages and their precursor hematopoietic stem and progenitor cells (Akbar et al., 2021). TNF-α stimulation increases the number of exosomes secreted from GMSCs and enhances exosomal expression of CD73, thereby inducing anti-inflammatory M2 macrophage polarization (Nakao et al., 2021). DPSC-Exos inhibit CD4^+^ T-cell differentiation into T helper cells and induce the transformation of these cells into regulatory T cells, thus elevating the levels of anti-inflammatory factors and playing an immunomodulatory role. In addition, scholars constructed a rat model of bisphosphonate-related osteonecrosis of the jaw and demonstrated that MSC-EVs prevented aging in stem cells, osteoblasts and fibroblasts induced by zoledronic acid and reduced the generation of inflammatory cytokines, thereby preventing bisphosphonate-related jaw necrosis (Watanabe et al., 2020). Moreover, because of their powerful anti-inflammatory properties, EVs have the ability to promote angiogenesis in an inflammatory environment. Studies have shown that inflammation may increase the number of exosomes secreted by PDLSCs. Exosomes released from inflamed PDLSCs promote angiogenesis in HUVECs by upregulating the expression of the vascular-specific marker CD31 and vascular endothelial growth factor (Zhang et al., 2020).

## 4 Conclusion and prospects

EVs are important factors for cellular communication and material transmission. In regenerative medicine, MSC-EVs have the potential to repair and regenerate oral tissue (Shi et al., 2020), including dental tissue, periodontal tissue, the maxilla and mandible, joint cartilage, maxillofacial soft tissue, peripheral nerves and other important structures. In addition, the source and state of donor cells play important roles in stem cell differentiation and tissue regeneration (Huang et al., 2016). For example, studies have shown that DPMSCs and BMMSCs have therapeutic effects in a rat stroke model, but the decrease in infarct volume in the DPMSC treatment group was more obvious than that in the BMMSC group (Song et al., 2017). The contents of EVs depend on the parent cell type, thus we supposed that EVs derived from these two stem cells might also have different therapeutic effects on stroke. However, aging is an unfavorable factor in mesenchymal stem cells from the perspective of cell-based treatment. The decreased function of aged stem cells is related to the attenuation of tissue regeneration. DPMSCs from young donors are more resistant to apoptosis than those from older donors and exhibit higher nonhomologous end-joining DNA repair activity (Zhai et al., 2017), which may explain why dental pulp stem cells from deciduous teeth are some of the most widely studied cells in oral tissue repair and regenerative medicine.

However, there are also many limitations and challenges in the clinical application of EVs for regenerative treatments. First, an effective method of isolating and purifying EVs from their parent cells or liquid has not been developed. At present, EVs are primarily extracted by ultracentrifugation, immunoadsorption, precipitation or microfluidic separation (Koritzinsky et al., 2017), all of which are cumbersome and error-prone. Therefore, applying these vesicles clinically is difficult due to the low extraction levels. Second, there is still a lack of horizontal comparisons of EVs in tissue repair and regeneration. For example, what is the reparative effect of EVs from different MSCs or what are the differences in composition and function between EV and CM from the same cell remain to be determined in widespread clinical studies. Third, a simple and controllable approach for specifically transporting EVs to injured sites is required for clinical application of EVs.

The combination of EVs and biomaterials has also become a popular research focus. For example, Diomede et al. combined a three-dimensional printed scaffold of PLA and human GMSCs-EVs to promote bone healing in rat skull defects (Diomede et al., 2018). Nooshabadi et al. fabricated a chitosan-glycerol hydrogel loaded with exosomes isolated from human endometrial stem cells, which was used for wound treatment in a mouse model (Nooshabadi et al., 2020). Ideal biomaterials always have superior performance and enhance the effects of extracellular vesicles to a certain extent, thus, finding new materials to combine with EVs for tissue damage repair must be a hot area of research in the future.
